# Editorial: Plant RNA structure

**DOI:** 10.3389/fpls.2023.1204600

**Published:** 2023-05-26

**Authors:** Huakun Zhang, Betty Y.-W. Chung, Zhiye Wang, Yiliang Ding

**Affiliations:** ^1^ Key Laboratory of Molecular Epigenetics of the Ministry of Education, Northeast Normal University, Changchun, China; ^2^ Department of Pathology, University of Cambridge, Cambridge, United Kingdom; ^3^ State Key Laboratory of Plant Physiology and Biochemistry, College of Life Sciences, Zhejiang University, Hangzhou, China; ^4^ Department of Cell and Developmental Biology, John Innes Centre, Norwich Research Park, Norwich, United Kingdom

**Keywords:** RNA structure, internal ribosomal entry site activity, *in vivo* RNA structure probing, thermoregulation, RNA G-quadruplex structure, plant growth and development, crop breeding, viroid

RNA folding is an inherent characteristic of RNA that plays a crucial regulatory function in all stages of post-transcriptional gene expression (Zhang and Ding, 2021). This Research Topic on Plant RNA Structure derives from the online Plant RNA Structure symposium in September 2021, where three main themes were discussed: 1) RNA structure functionality in plant response to environmental changes; 2) Noncoding and viral RNA structure in plants; 3) Evolution and adaptation of RNA structure in plants.

Recent developments in both technology and scientific knowledge have significantly promoted the Plant RNA Structure research field. This Research Topic lists two research articles and four mini-reviews with peer review. It aims to summarise the latest advances in plant RNA structure studies, emphasizing the biological significance of RNA structure and outlining potential future research directions.

Technological breakthroughs in studying RNA secondary and tertiary structures have improved our understanding of RNA structure functionality in plants. For RNA secondary structure profiling, several chemical-based profiling methods were developed in plants (Jaramillo-Mesa et al.; Jin et al.; Sun et al.). Dimethyl sulfate (DMS) is a commonly used chemical that reacts with the Watson-Crick faces of single-stranded Adenine and Cytosine nucleotides (Jin et al.). Another chemical-based profiling method is selective 2′-hydroxyl acylation analysed by primer extension (SHAPE) which detects the single-strandedness by acetylating the 2′-OH group of accessible RNA nucleotides. One widely used SHAPE chemical reagent is 2-methylnicotinic acid imidazolide (NAI) (Jaramillo-Mesa et al.; Sun et al.). Jin et al used DMS mutational profiling with high-throughput sequencing (DMS-MaPseq) and profiled *in vivo* RNA secondary structures in rice (*Oryza sativa*) at both target-specific and genome-wide scales. Their work revealed *in vivo* RNA structure features of *OsPHO2*, a key regulator of inorganic phosphate (Pi) homeostasis and the phosphate-starvation response pathway. Notably, the 5’UTR of *OsPHO2* contains the OsmiR399 target site, where a single-stranded structure immediately downstream of the miRNA target site was observed, suggesting a regulatory function of mRNA structure on miR399-mediated Pi homeostasis. This demonstration of RNA secondary structure features under the phosphate-starvation condition in rice shows promise for future investigations of RNA structure in rice and other crops in response to nutrition deficiency.

SHAPE has been widely used to study RNA structure functionality (Jaramillo-Mesa et al.,; Liu et al.,; Sun et al.,). Jaramillo-Mesa et al. used SHAPE to validate the internal ribosomal entry site (IRES) structure in Triticum mosaic virus (TriMV). This guided the design of anti-sense oligonucleotides in repressing viral translation. Moreover, this work showed the role of IRES core modular RNA domains and their potential “free-for-interaction” status in both ribosomal recruitment and start codon selection in a multi-AUG viral 5’UTR. These mechanisms involving structural changes in RNA facilitate translational regulation in plants. The RNA structure profiling techniques used in this study allowed to investigate the secondary structure characteristics of plant viral RNAs, an area where earlier research was predominantly restricted to *in silico* and *in vitro* studies. The minireview of Ma and Wang provides an overview on RNA structure studies applied to viroids and how tertiary structural motifs in RNA were systematically identified and shown to have important roles in the mediation of viroid movement, including nuclear import and trafficking across diverse cellular boundaries in plants.

The review by Sun et al. describes genome-wide studies on RNA structure in crops. Not only SHAPE but also DMS and potentially also other methods were used (Sun et al.). The authors also propose potential crop molecular breeding strategies based on the RNA structure-associated regulation of gene expression, such as translation and RNA degradation (Sun et al.). In addition, they suggest that riboSNitches, as single nucleotide variants (SNVs) resulting in RNA structure disparities, may offer new ways for interpreting the results of genome-wide association studies (GWAS) (Sun et al.). Moreover, riboSNitches may offer potential as molecular markers for breeding line selection to boost future molecular breeding endeavours.

As sessile organisms, plants are comparatively more prone than other eukaryotes to the effects of environmental conditions such as changes in temperature (Thomas et al.). Notably, most of these environmental conditions influence RNA structure dynamics (Thomas et al.). The authors comprehensively compared RNA secondary structure thermosensitivities between prokaryotes and eukaryotes, highlighting the role of RNA structure in mediating thermoregulation in plants. The RNA structure motif termed RNA G-quadruplex (RG4) previously known to provide thermotolerance of RNA to cold emerged as a new type of thermosensor regulating plant responses to temperature (Liu et al.). In their minireview, Liu et al. summarize technological advances, including both chemical profiling and cell imaging, for determining the RG4 folding status in living cells. The authors also thoroughly compare different methods for RG4 detection and summarize novel functions of RG4s in regulating RNA stability and translation and, thus, plant growth and development at different temperatures. The authors suggest that the evolution of RG4 folds contributed to plant evolution and plant adaptation to the habitats found across the plant kingdom.

The current dynamic and rapid advances in the plant RNA structure field is illustrated by the wide-ranging articles included in this Research Topic. Improving comprehension of the functional significance of RNA structure in various plant biological processes will usher a new era of research on the regulation of gene expression by modifications in RNA structure. By enhancing the research capacity for conducting genome-wide RNA structure studies across extensive plant species and viral RNAs, this progressive understanding will also facilitate the identification of both conserved and divergent RNA structures among natural variants and diverse plant species. Such studies are expected to offer opportunities for investigating the role of RNA structure in plant evolution and habitat selection. Technological advances of RNA structure probing coupled with third generation high-throughput sequencing platforms, such as Pacbio and Nanopore, will achieve high-resolution RNA structure information e.g., at single-molecular and single-cell levels. Further investigations into plant RNA structure dynamics under diverse abiotic and biotic stresses are likely to reveal more RNA structure-mediated regulations in plant responses to environmental changes. Insights into the role of RNA structure in plant thermotolerance and in thermosensing to regulate gene expression has great potential for crop enhancement strategies to mitigate the global impact of climate change on yield. The accelerating study of RNA structures will enable the scientific community to tackle these and other challenges rapidly in upcoming years, offering critical and exciting new insights into our comprehension of RNA structure-mediated regulatory mechanisms in plants.

## Author contributions

All authors listed have made a substantial, direct, and intellectual contribution to the work, and approved it for publication.
